# A reliable unmanned aerial vehicle multi-ship tracking method

**DOI:** 10.1371/journal.pone.0316933

**Published:** 2025-01-10

**Authors:** Guoqing Zhang, Jiandong Liu, Yongxiang Zhao, Wei Luo, Keyu Mei, Penggang Wang, Yubin Song, Xiaoliang Li

**Affiliations:** 1 North China Institute of Aerospace Engineering, Langfang, China; 2 Aerospace Remote Sensing Information Processing and Application Collaborative Innovation Center of Hebei Province, Langfang, China; 3 National Joint Engineering Research Center of Space Remote Sensing Information Application Technology, Langfang, China; King Fahd University of Petroleum & Minerals, SAUDI ARABIA

## Abstract

As the global economy expands, waterway transportation has become increasingly crucial to the logistics sector. This growth presents both significant challenges and opportunities for enhancing the accuracy of ship detection and tracking through the application of artificial intelligence. This article introduces a multi-object tracking system designed for unmanned aerial vehicles (UAVs), utilizing the YOLOv7 and Deep SORT algorithms for detection and tracking, respectively. To mitigate the impact of limited ship data on model training, transfer learning techniques are employed to enhance the YOLOv7 model’s performance. Additionally, the integration of the SimAM attention mechanism within the YOLOv7 detection model improves feature representation by emphasizing salient features and suppressing irrelevant information, thereby boosting detection capabilities. The inclusion of the partial convolution (PConv) module further enhances the detection of irregularly shaped or partially occluded targets. This module minimizes the influence of invalid regions during feature extraction, resulting in more accurate and stable features. The implementation of PConv not only improves detection accuracy and speed but also reduces the model’s parameters and computational demands, making it more suitable for deployment on computationally constrained UAV platforms. Furthermore, to address issues of false negatives during clustering in the Deep SORT algorithm, the IOU metric is replaced with the DIOU metric at the matching stage. This adjustment enhances the matching of unlinked tracks with detected objects, reducing missed detections and improving the accuracy of target tracking. Compared to the original YOLOv7+Deep SORT model, which achieved an MOTA of 58.4% and an MOTP of 78.9%, the enhanced system achieves a MOTA of 65.3% and a MOTP of 81.9%. This represents an increase of 6.9% in MOTA and 3.0% in MOTP. After extensive evaluation and analysis, the system has demonstrated robust performance in ship monitoring scenarios, offering valuable insights and serving as a critical reference for ship surveillance tasks.

## 1. Introduction

Ship monitoring technology is extensively utilized across various domains, including maritime vessel surveillance, shipping regulation, and search and rescue operations. Despite its widespread application, real-world challenges such as variable lighting conditions, complex ocean backgrounds, small target sizes, occlusions, weather disturbances, and considerable intra- and inter-class variations complicate the tasks of ship detection and tracking [1–3]. These factors necessitate heightened accuracy and real-time capabilities in ship monitoring systems. The rapid development of computer vision and unmanned aerial vehicle (UAV) technologies has broadened their application to include ship detection and tracking [4], thus pioneering new avenues in maritime surveillance.

Ship monitoring methodologies are categorically divided into traditional and computer vision-based approaches [5]. Traditional methods, such as manual observation from watchtowers, though straightforward and intuitive, suffer from limitations in coverage, continuous monitoring, and operational efficiency. Prolonged periods of operation may also lead to operator fatigue, thereby heightening safety risks [6]. To overcome these challenges, computer vision-based methods [7,8] have been developed, facilitating automated ship identification systems (AIS) that utilize remote sensing imagery for more accurate and efficient ship detection. For instance, Wu et al. employed the YOLOv3 model to achieve rapid and precise detection of ships in optical remote sensing images, integrating a multi-granularity network (MGN) to enhance the extraction of target appearance features from SAR images. This approach was further refined by incorporating multiscale features with an attention mechanism to improve model discrimination [9]. Similarly, Song et al. [10] introduced a technique to detect ships in hazy ocean remote sensing images by applying color polarization classification and haze concentration clustering, which help balance the colors of the images and reduce haze interference. Additional algorithms such as R3Det [11], S2Anet [12], Light-SDNet [13], and ARS-Det [14] have also been implemented for ship detection in remote sensing imagery. Nonetheless, AIS based on ship remote sensing data continues to face significant challenges, including a lack of data diversity and limited multi-angle data samples. UAVs, recognized for their high flexibility, have been extensively applied in various sectors. Employing UAVs for data collection can effectively address the issue of data diversity, positioning them as a highly promising solution for data acquisition in the context of ship monitoring.

In recent years, UAVs have been extensively utilized in the field of ship monitoring, playing pivotal roles in vessel traffic flow prediction [15], speed measurement [16], and maritime surveillance [17]. UAVs serve as versatile and intelligent tools for ship recognition, monitoring, and tracking across various maritime scenarios. They are capable of operating over wide areas under diverse weather and lighting conditions without geographical limitations [18], which underscores their efficiency and broad applicability in maritime surveillance. Additionally, UAVs can be equipped with a range of sensors, including optical cameras, radar, infrared, and sonar, to gather multifaceted information about ships from different perspectives, significantly enhancing the accuracy and reliability of ship recognition and tracking [19]. Furthermore, UAVs have the capability to transmit images and data in real time to ground control centers or to other UAVs, facilitating multi-UAV cooperative operations [20] and improving the flexibility and coordination of maritime surveillance efforts. Compared to traditional manned aircraft or satellites, UAVs offer enhanced safety and reduced manufacturing and operational costs, which diminish the risks and resource consumption associated with maritime surveillance. However, UAV-based ship tracking continues to confront numerous challenges, such as complex ocean backgrounds, dynamic ship movements, variations in illumination, and changes in target appearance.

The integration of AI technology with UAV technology is regarded as crucial for addressing these challenges. Various advanced techniques, such as the refined YOLOv7 algorithm [21], SSMA-YOLO [22], and YOLO-Vessel [23], have been developed for detecting ships in aerial images captured by UAVs. For instance, Wang et al. introduced a network architecture named GGT-YOLO specifically for detecting ships in UAV-taken aerial images [24]. Similarly, Chen et al. devised a hybrid approach that combines an optimized generative adversarial network (GAN) with a convolutional neural network (CNN)-based detection system to achieve high-precision detection and identification of small ships in complex maritime environments [25]. Furthermore, Zhao et al. developed YOLOv7-sea, an algorithm designed to detect tiny objects on the sea surface. This detector identifies regions of interest in maritime scenes by adding a predictive head and incorporating a parameter-free attention module [26]. While these UAV-based ship detection algorithms have shown remarkable results in terms of detection accuracy, they still face limitations in predicting ship trajectories and tracking dynamic changes in ship movement.

In the realm of maritime surveillance and situational awareness, Multi-Object Tracking (MOT) is a critical task that provides essential information about ship location, identity, and movement [27,28]. Within MOT, algorithms such as SORT [29], Deep SORT [30], BoT SORT [31], ByteTrack [32], and MOTDT [33] enable end-to-end detection and tracking autonomously by extracting advanced features from UAV images [34]. However, these MOT methodologies often fall short in complex and densely populated maritime environments. They struggle with issues such as false positives, missed detections, and tracking failures, which are frequently exacerbated by dynamic ship movements, variations in lighting and appearance, and occlusions. To address these challenges, this study introduces a multi-ship tracking system that leverages the capabilities of YOLOv7 combined with the Deep SORT algorithm, integrating unmanned aerial vehicle technology and deep learning. By enhancing both the YOLOv7 and Deep SORT models, this system achieves high-precision and real-time ship monitoring. The principal contributions of this research are outlined as follows:

1. The YOLOv7 model incorporates transfer learning to mitigate the challenges associated with limited training data, high dataset preparation costs, and substantial labor expenses, thereby improving detection accuracy and accelerating network convergence.
2. The integration of the SimAM attention mechanism into the YOLOv7 detection framework significantly enhances object detection accuracy by directing the algorithm’s focus towards relevant image features.
3. The adoption of PConv optimizes the multi-scale feature fusion module, which enhances detection speed while reducing the number of parameters and computational effort required.
4. During the IOU matching stage of the Deep SORT algorithm, DIOU replaces IOU for matching between unsuccessful trajectory and detection results. This adjustment effectively reduces false negatives caused by target aggregation or occlusion, thereby enhancing both target trajectory tracking and matching accuracy.

The content of this article is organized as follows: section 2 introduces the relevant work of this study; section 3 describes the dataset and research methods; section 4 introduces and discusses the experimental results. Finally, section 5 summarizes the conclusions of the work.

## 2. Related work

In recent years, deep learning-based methods for ship monitoring have garnered significant attention, with the YOLO series of models demonstrating particularly impressive performance in ship detection. Tang et al. [35] introduced the H-YOLO model, which achieved a 19.01% improvement in recognition rate and a 16.19% increase in accuracy by pre-selecting regions of interest and differentiating color contrasts between ships and their backgrounds. Despite these advancements, the high computational demands of H-YOLO render it unsuitable for real-time applications. Jiang et al. [36] proposed the YOLOv4 light model, which maintains a high recognition accuracy of 90.37% while simplifying the model’s structure, although some redundancies still exist. The LiftYOLOv5 model, developed by Xu et al. [37], employs histogram background classification and shape distance clustering modules to enhance recognition accuracy by 1.51% and reduce the number of parameters. Yao et al. [38] significantly increased the accuracy of multi-class ship detection using the YOLOv8 model; however, the limited dataset coverage resulted in a scarcity of training samples for large ships. Zhao et al. [39] proposed the YOLOv7-sea model, which improves the focusing capability on target areas through the introduction of an attention mechanism, yet it still faces challenges in multi-scale feature extraction. To address these limitations in feature extraction, dynamic convolution technology has been progressively integrated into ship monitoring tasks. For instance, the ODConv (Full Dimensional Dynamic Conv) [40] markedly enhances detection performance by dynamically adjusting the convolution kernel. Additionally, Cheng et al. [41] incorporated dynamic convolution modules into shallow networks, boosting recognition efficiency in complex backgrounds. Nevertheless, challenges such as false positives and extended training durations persist in the detection of small vessels.

Background interference and noise in complex marine environments continue to pose significant challenges for ship monitoring. To mitigate these issues in intricate scenarios, Zhao et al. [42] developed a YOLOv8 model integrated with the lightweight MobileViTSF, enhancing the precision of ship target detection against complex backgrounds. Nonetheless, the model’s detection accuracy still falls short in scenarios with densely packed ships. Park et al. [43] assessed various YOLO-based models on the Singapore Maritime dataset and introduced an IoU-distance-based online maritime multi-target tracking method. However, this method did not successfully resolve the issues of target occlusion. Han et al. [44] merged the Single Shot Multibox Detector (SSD) with the Extended Kalman Filter (KF) to improve the detection and tracking of surface unmanned vehicles. Despite these advancements, the loss of features from long-distance targets remains a considerable challenge in complex environments. Similarly, Lee et al. [45] developed a perception system employing YOLOv3 coupled with KF, which still struggles with capturing long-distance target information. To enhance multi-ship tracking under occlusion conditions, Liu et al. [46] incorporated SIFT features into the DeepSORT model. Meanwhile, Ding et al. [47] enhanced detection and tracking accuracy using YOLOv5 and DeepSORT on publicly available ship datasets. Guo et al. [48] significantly boosted the tracking stability of nonlinear moving targets on the sea surface with an enhanced YOLOv7 and C-BIoU algorithm. Continuing this effort, Guo et al. [49] proposed DeepSORVF, a simple online real-time ship data fusion method based on deep learning, aimed at high-precision detection and tracking of ships. However, in complex environments like crowded waterways or ports, rapid target movement and occlusion still lead to tracking failures. Wang et al. [50] proposed an improved YOLOv7 model for high-precision ship detection and recognition, yet it did not address the issues of misdetection and missed detections due to target occlusion. Most of these studies rely on publicly available datasets or fixed cameras, which suffer from insufficient data diversity and a limited field of view, challenging comprehensive monitoring capabilities. Moreover, the ship detection and tracking process frequently encounters problems such as misdetection, omissions, and lack of precision. In contrast, this study has collected a diverse array of ship data using equipment equipped with UAVs, offering flexibility and an expansive field of view, effectively compensating for the lack of data diversity. Furthermore, this study proposes an intelligent ship monitoring system based on the MOT method, specifically designed to address the challenges of complex marine environments and capable of achieving high-precision and real-time ship monitoring.

## 3. Material and method

### 3.1 Datasets

This study utilized ship videos captured by UAVs as the dataset. We selected 20 ship video sequences, dividing them into two sets: 10 for target detection and 10 for target tracking. These datasets served as benchmarks for evaluating ship detection and tracking performance using the pre-trained YOLOv7 model and the Deep SORT algorithm.

To achieve a pre-trained model with high detection accuracy and rapid network convergence, the YOLOv7 model was pre-trained on the open ship dataset known as the "seaships" set, which comprises a total of 7,000 images. These images were split into training and test sets at a ratio of 9:1.

For effective tracking and monitoring using the Deep SORT algorithm, a substantial dataset is necessary to extract appearance features of ships. The DarkLabel software was employed to annotate these images, storing them in XML format, which forms the basis of the target tracking dataset. The dataset was divided into training, testing, and validation subsets at ratios of 70%, 20%, and 10%, respectively.

### 3.2 Detector

The limited number of datasets for ship object detection poses challenges in obtaining comprehensive training outcomes directly applicable for training the YOLOv7 model. Specifically, the convolution layers in the feature extraction segment require extensive training to discern discriminative features from the target detection dataset. Consequently, transfer learning was employed in this study to enhance the YOLOv7 model’s detection accuracy and network convergence speed, addressing issues related to the scarcity of training data, high data preparation costs, and labor intensity.

**Fig 1:**
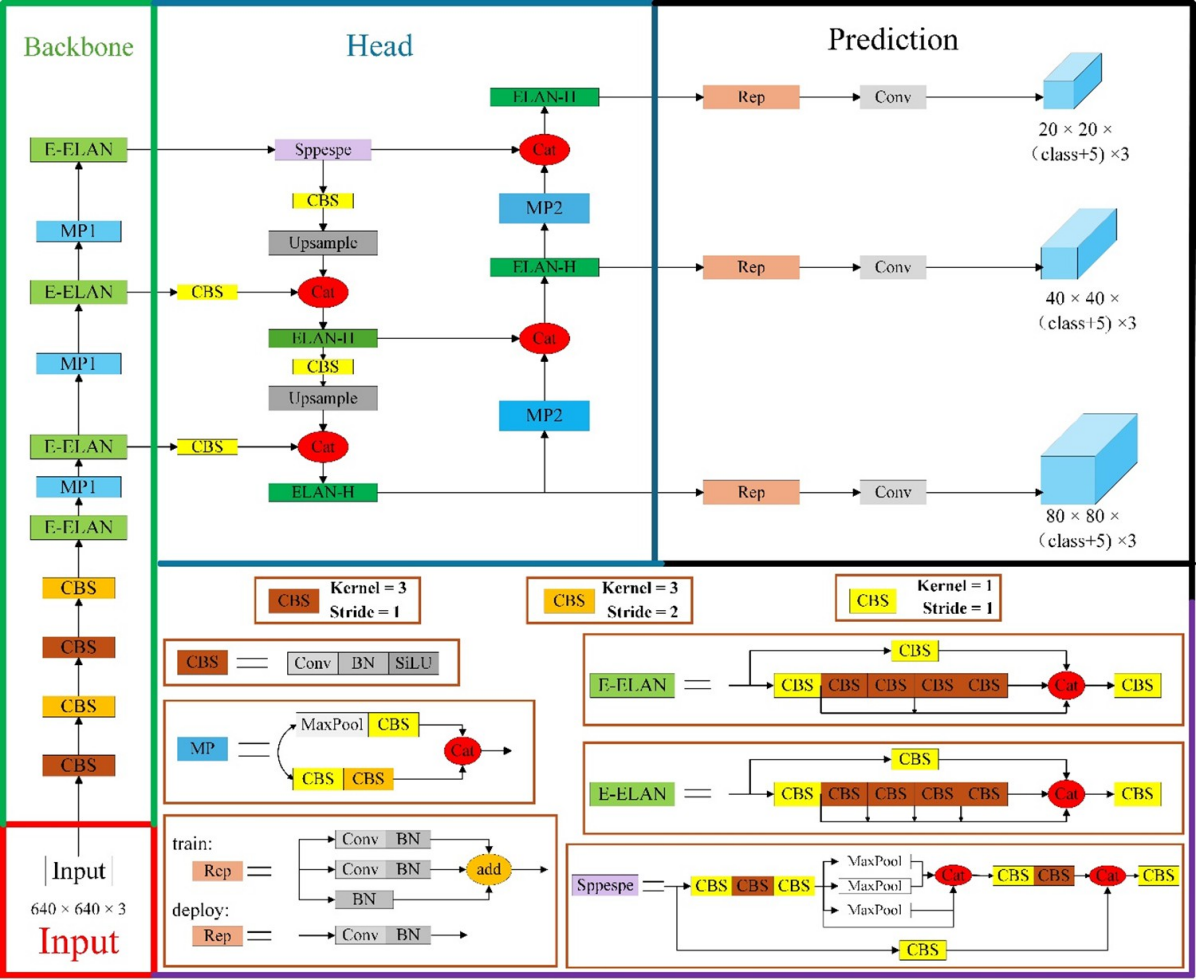
Network structure of the YOLOv7 model.

The YOLOv7 model [51], developed in 2022 by Chien-Yao Wang and Alexey Bochkovskiy, is designed to balance detection efficiency and accuracy effectively. It integrates the Extended High-Efficiency Layer Aggregation Network (E-ELAN) [52] with strategies such as model scaling and model re-parameterization [53], based on cascading models [54]. As depicted in Fig 1, the YOLOv7 network architecture includes four distinct modules: the Input module, the Backbone network, the Head network, and the Predict network head.

#### 3.2.1 Object detection using YOLOv7 with transfer learning

**Fig 2:**
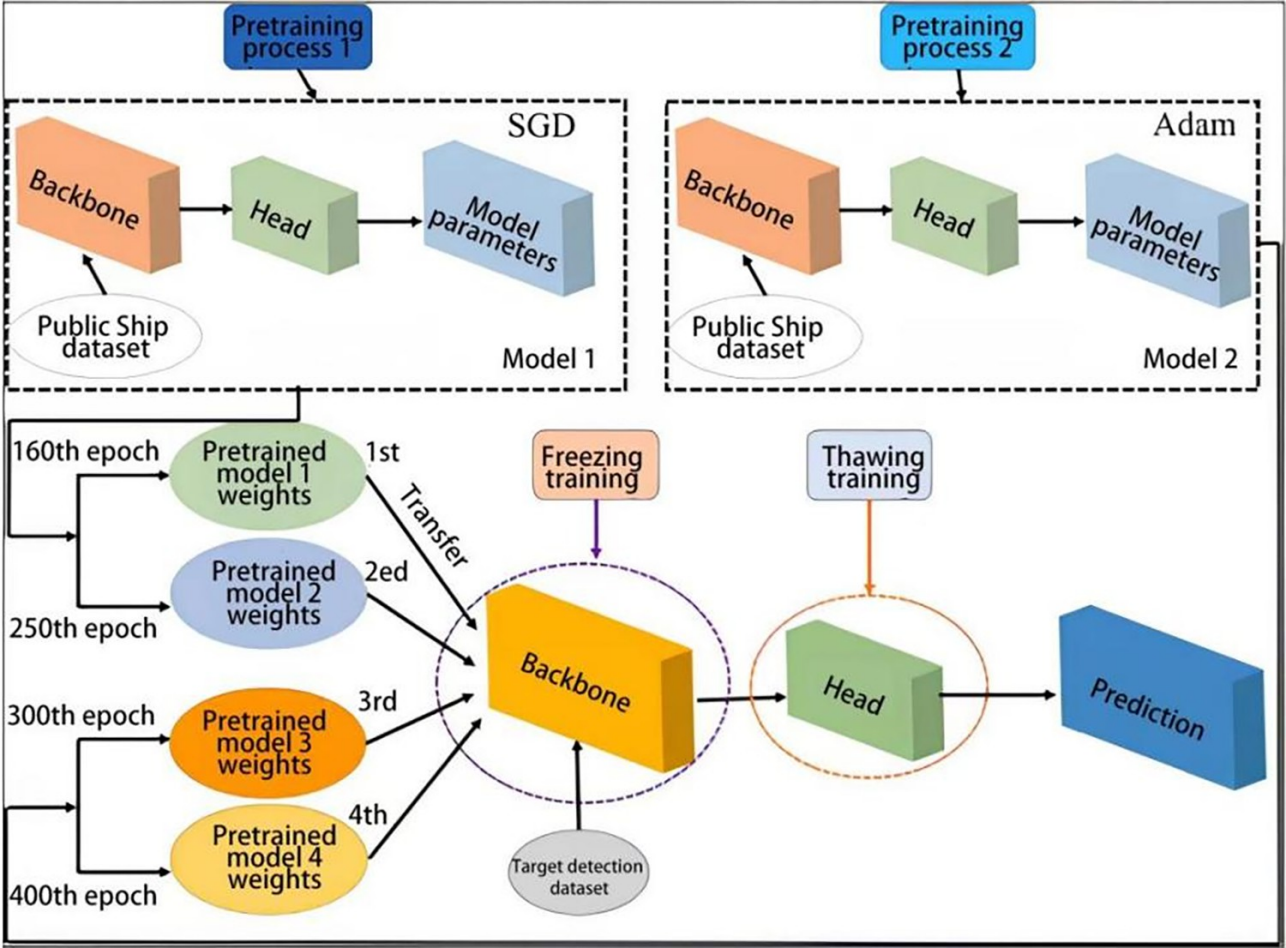
Schematic diagram of the transfer learning process.

In this study, we employed a model parameter-based transfer learning approach with fine-tuning of the YOLOv7 network weights for training. During the pre-training phase on a publicly available ship dataset, YOLOv7 models equipped with two distinct optimizers—SGD and Adam (referred to as Model 1 and Model 2, respectively)—were utilized to expedite gradient reduction and attain a globally optimal solution. These optimizers exhibit fundamentally different optimization algorithms and training convergence behaviors compared to other optimizers. The iteration numbers of the models were used as key parameters in the training process. Specifically, the parameters at the 160th and 250th iterations for Model 1, and the 400th and 300th iterations for Model 2, were designated as Pre-trained Models 1 to 4, respectively. Following the transfer learning phase and the training of these pre-trained models’ weight parameters, the resultant detection models were named Transfer Learning Models 1–4. These models were subsequently employed to detect and identify ships in the target detection dataset, with the best-performing model selected for real-scene ship tracking identification. Table 1 lists the hyperparameter settings of the models, and Fig 2 illustrates the schematic diagram of the training process using the transfer learning method.

**Table 1:** Hyperparameters of the pre-trained model.

Hyperparameters	Names and values	
Optimizer	SGD	Adam
Momentum	0.973	0.973
Weight decay	5×10^−4^	0
Maximum learning rate	1×10^−2^	1×10^−3^
Minimum learning rate	1×10^−4^	1×10^−5^
Epoch	300	400
Batch size	4	4
GPU	NVIDIA Volta	NVIDIA Volta
Library	Pytorch	Pytorch

Due to the high similarity between the features of the open ship dataset and the target detection dataset, a combination of freezing and thawing methods was deemed appropriate for training. The YOLOv7 network, enhanced through transfer learning, not only accelerates training and convergence speeds but also maximizes computational efficiency, thereby improving the training effectiveness and accuracy of the model while preventing weight degradation. Fig 2 outlines the primary steps of the transfer learning training process for the YOLOv7 model, which can be described as follows:

1. Models 1 and 2 are pre-trained separately on the pre-training dataset.
2. The iteration numbers of Models 1 and 2 serve as transfer parameters, selecting four significant periods for migration learning.
3. During this phase, the existing weights—primarily adjusted to the target detection dataset—are employed to freeze the backbone sections of Models 1 and 2 while unfreezing the head sections. The transferred parameters from Models 1 and 2 are then integrated into the backbone of the newly pre-trained models, which undergo simultaneous training with the target detection dataset in a frozen state. Subsequently, the parameters of the backbone sections are transferred and further training is conducted.
4. Initially, the target detection dataset is in a frozen state. Subsequently, the target object task is used as the output to lift the freeze.
5. After completing the freeze training and transferring the feature information to the head network, the unfrozen training is conducted.
6. Finally, the transfer learning detection model is obtained after the learning and training of weight parameters from the pre-trained models are completed.

#### 3.2.2 SimAM

**Fig 3:**
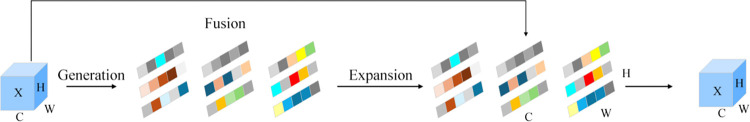
SimAM structure.

Given the objective of this study to monitor ships, particularly when many are partially occluded, it is crucial to enhance the feature details in regions where occlusion occurs. This emphasis ensures that even partially obscured objects remain identifiable, thereby increasing the accuracy of detection and laying a foundation for subsequent tracking of such targets. To achieve this, we introduce an efficient attention module for convolutional neural networks, termed SimAM. Unlike traditional channel and spatial attention modules, which require additional parameters within the network, SimAM calculates the 3D attention weights directly from the feature maps of a specific layer, and it also accounts for targets that are partially obscured. This module is seamlessly integrated into the head section of the YOLOv7 architecture without modifying the network’s primary structure. The adapted YOLOv7 architecture incorporating the SimAM module is illustrated in Fig 3.

Current methods in attention mechanisms typically compute weights in one or two dimensions from a feature matrix, denoted as X. These weights are then uniformly applied either across the channel or spatial dimensions. In channel attention, which operates on one dimension, differentiation occurs among channels while treating all spatial locations identically. Conversely, spatial attention, operating in two dimensions, distinguishes among various spatial locations but applies a uniform treatment across all channels. This method limits their ability to identify more nuanced discriminative features. By assigning three-dimensional weights to the network channels, SimAM significantly reduces these limitations and enhances performance compared to traditional one-dimensional and two-dimensional attention mechanisms. The SimAM attention module autonomously computes these 3D weights, ensuring consistent color assignment across all channels, spatial positions, and elemental points within subgraphs. The integration of the SimAM module results in a marked improvement in 3D feature extraction, thus elevating the accuracy of detection.

#### 3.2.3 ELAN_PC

**Fig 4:**
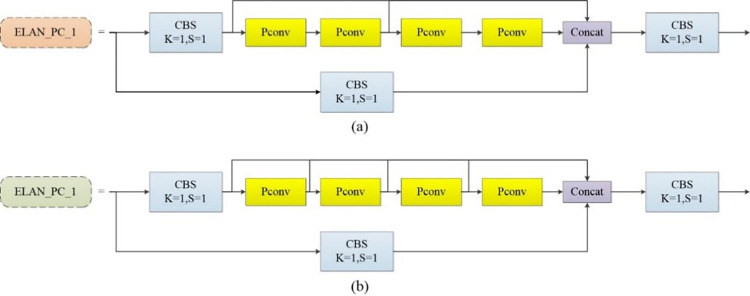
Improved ELAN structure.

ELAN [55] represents an advanced layer aggregation network that incorporates elements from VoDateTime [56] and CSPNet [57], aimed at enhancing feature extraction by optimizing both short and long gradient paths. The architecture of ELAN consists of two principal branches: the first branch adjusts channel numbers via 1×1 convolution, while the second branch also modifies channels with 1×1 convolution, followed by feature extraction through four 3×3 convolutional modules. Subsequently, it merges the outputs from both branches. This configuration is designed to mitigate issues of gradient vanishing in deeper layers. However, the performance of ELAN in terms of parameter count and computational efficiency remains suboptimal. To address these limitations, we have integrated the PConv layer into the network, resulting in the creation of the ELAN_PC_1 and ELAN_PC_2 modules. In these enhanced modules, the original 3×3 convolutional layers in ELAN are substituted with PConv layers. The designs of ELAN_PC_1 and ELAN_PC_2 are illustrated in Fig 4.

**Fig 5:**
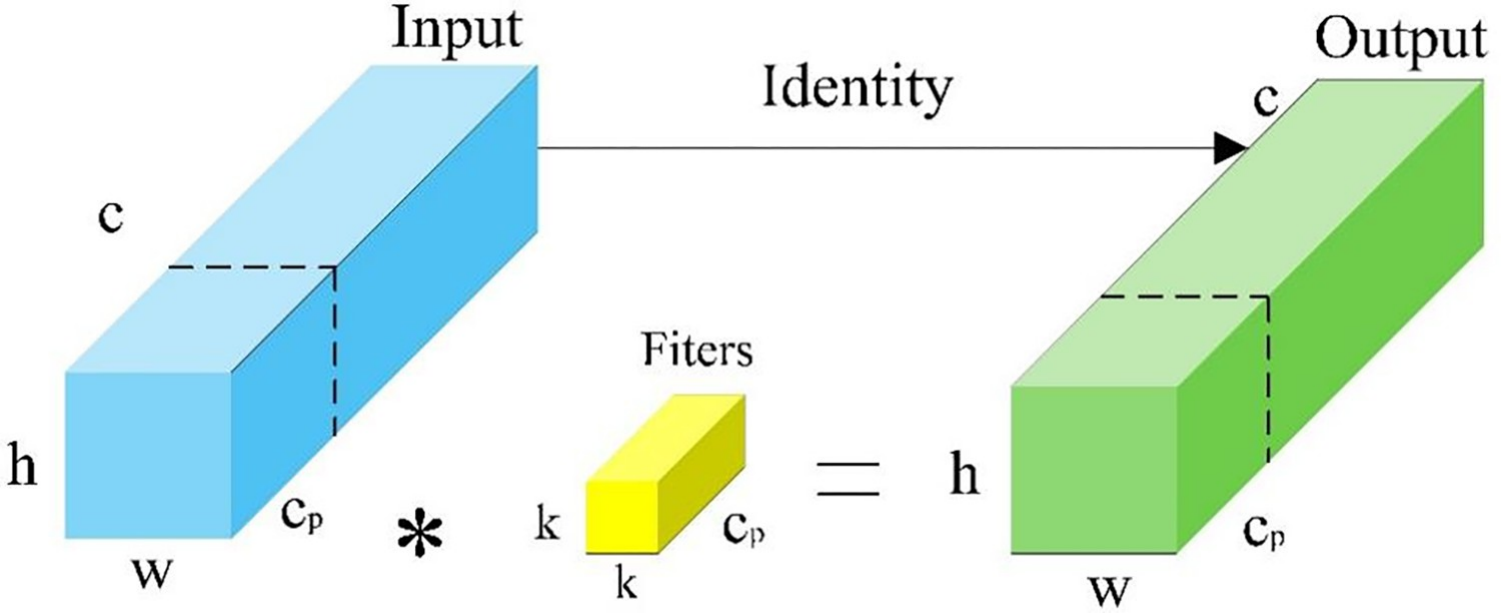
PConv module structure.

PConv [58] is an innovative, lightweight convolution module designed to reduce computational redundancy and decrease memory access requirements. This efficiency is achieved by performing convolution operations selectively on a subset of input channels, thereby minimizing unnecessary redundancy in the feature map. The structure of the PConv module is depicted in Fig 5.

For sequential or periodic memory accesses, the input or output channel *C_p_* represents the entire feature map. In PConv, the computation is calculated as $h\times w\times k^{2}\times c_{p}^{2}$, whereas for standard convolution, it is *h*×*w*×*k*^2^×*c*^2^. PConv requires memory access of *h*×*w*×2*c_p_*, compared to *h*×*w*×2*c* for standard convolution. When cp=c4, the computational cost of PConv is reduced to one-sixteenth that of regular convolution, and the memory access is reduced to one-fourth.

The integration of the PConv convolution module into the ELAN architecture significantly reduces computational demands and memory usage, leading to a lighter model with enhanced inference speeds.

### 3.3 Tracker

The Deep SORT algorithm was utilized as the foundational algorithm for the tracking system, with its IOU matching phase enhanced by substituting IOU with DIOU. This modification improves both the accuracy of matching and the trajectory estimation of the targets.

#### 3.3.1 Objects tracking method

The Deep SORT algorithm employs a KF to predict the movement of targets and integrates these predictions with target appearance features extracted using a deep CNN. This combination facilitates effective tracking of the targets.

**Fig 6:**
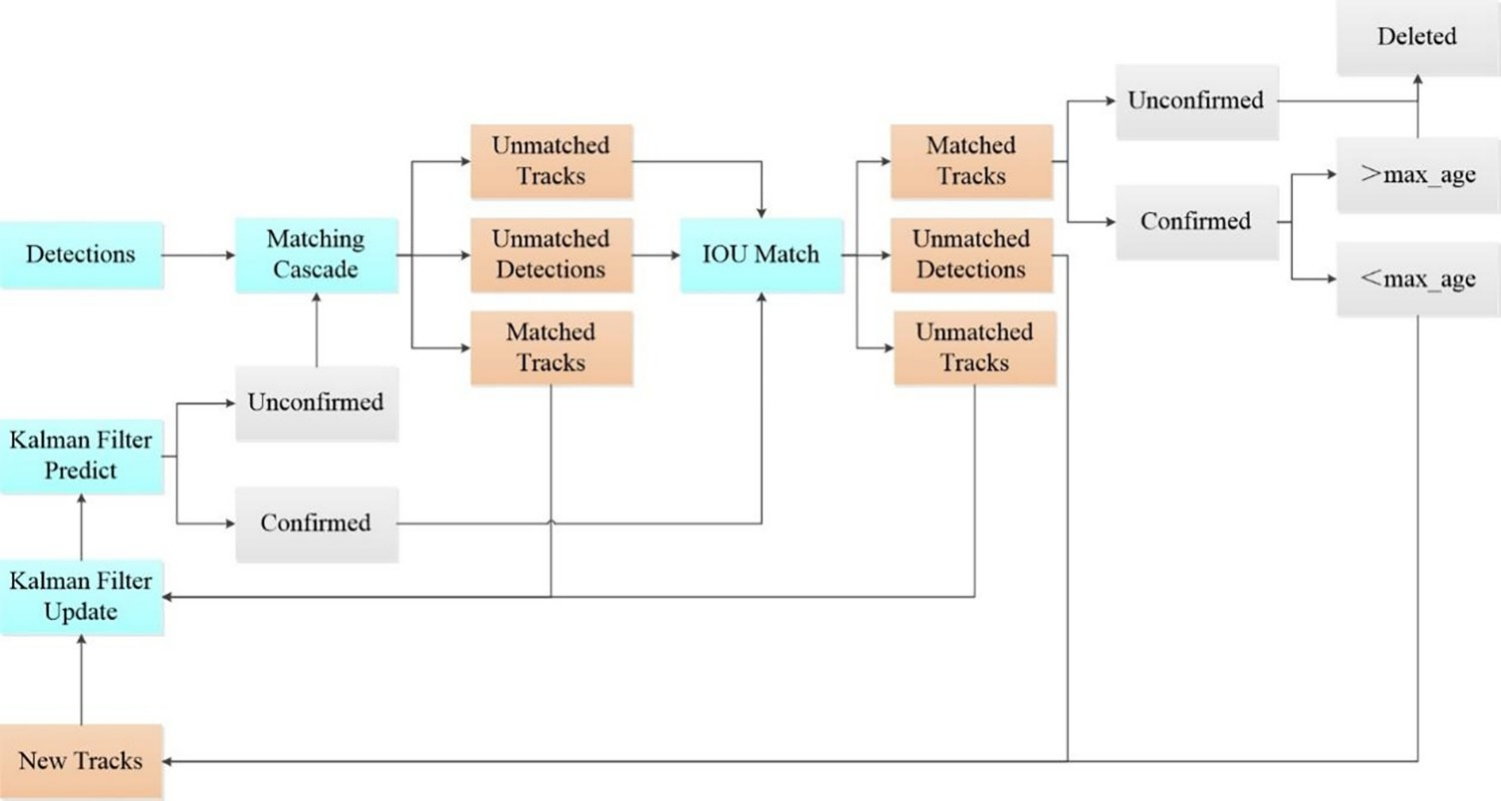
Deep SORT module structure.

Deep SORT extends the capabilities of the SORT algorithm by incorporating a similarity metric to evaluate the appearance features of targets, along with a cascade matching mechanism. These improvements significantly reduce identity switches, particularly when targets are occluded, thereby enhancing the robustness of the model. The original SORT algorithm utilizes a Kalman filter to predict the motion states of objects and employs the IOU metric for data association, which is determined by comparing the predicted target bounding boxes against those detected by the network. The associations are then finalized using the Hungarian algorithm, crucial for the continuous tracking of targets. The effectiveness of the tracking process heavily relies on the performance of the feature extraction network, which is essential for capturing detailed and accurate visual representations of the subjects. As illustrated in Fig 6, the architecture of the Deep SORT algorithm encompasses two main components: the deep appearance descriptor branch and the motion prediction branch. The motion prediction branch forecasts trajectory states through Kalman filtering, utilizing data from previous frames to predict the positions of targets in upcoming frames. Discrepancies between the predicted trajectories and actual detections are assessed using the Mahalanobis distance. Functioning as a straightforward convolutional network, the deep appearance descriptor branch performs image classification and extracts appearance features from detected frames into a vector of features. The cosine distance metric is used to assess the similarity between these feature vectors. Track segments are linked through a matching cascade algorithm that employs both cosine and Mahalanobis distances. During the track management phase, tracks are updated, initialized, and deleted, ensuring efficient tracking continuity.

#### 3.3.2 Improvement of IOU matching

In this work, the Deep SORT algorithm is enhanced by substituting the traditional IOU with DIOU for matching unsuccessful trajectories with detection results. The improved algorithm takes detection results as input, where trajectories are predicted using a KF, and these newly predicted trajectories are matched against the current detected video frame using the Hungarian algorithm. Successfully matching an unsuccessful trajectory with detection results leads to an update in the KF.

IOU is a metric characterized by its unity, symmetry, satisfaction of the triangle inequality, and spatial scale invariance. The formula for the IOU loss function, *L_IOU_*, is as follows:

$$L_{IOU}=1-S_{IOU}$$ (1)

**Fig 7:**
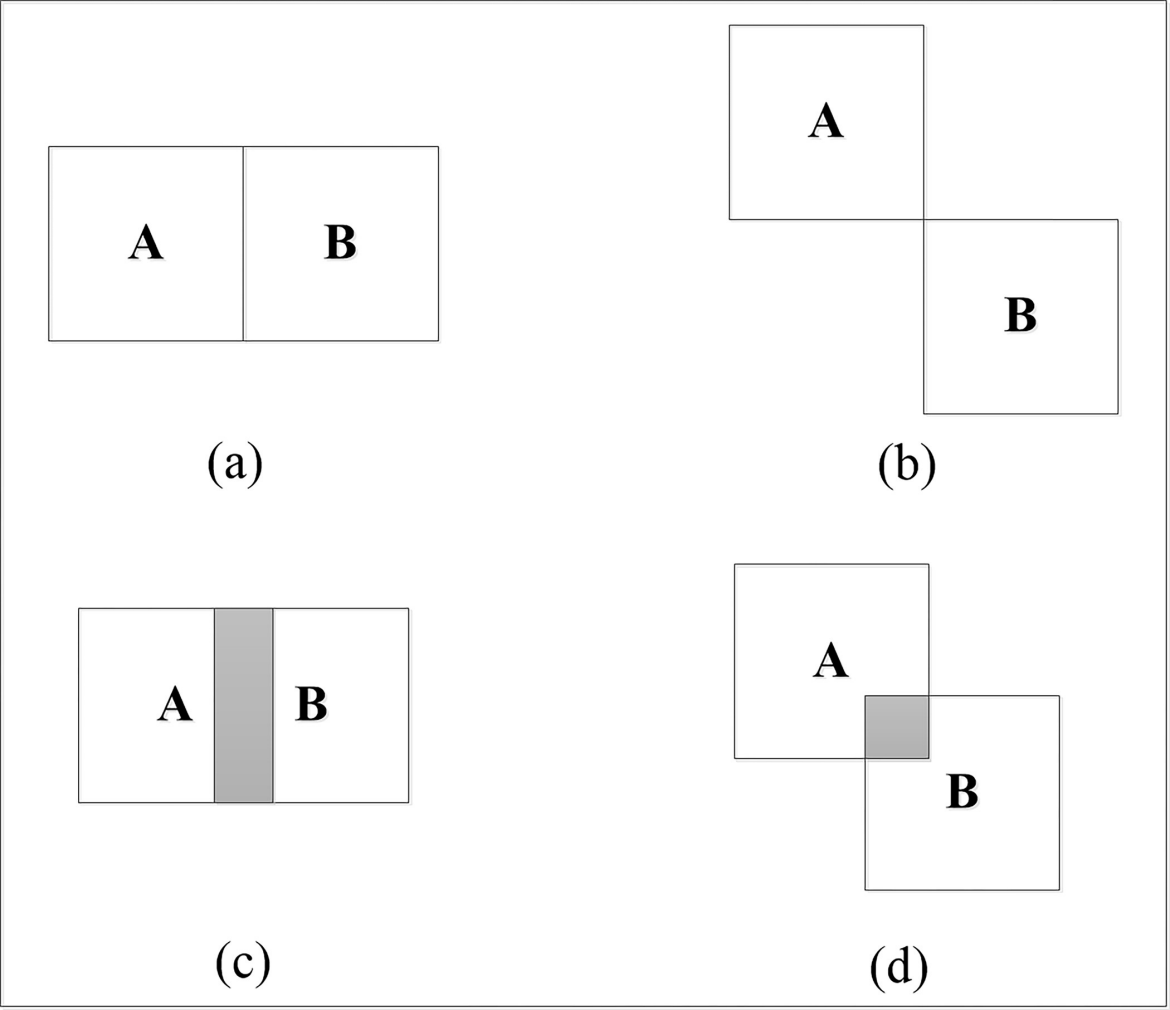
IOU status.

However, IOU does not provide information on the distance or the extent of intersection between detection frame A and predicted frame B as illustrated in Fig 7. In scenarios (a) and (b), both *S_IOU_* values are zero. The distance from A to B in Fig 7A is significantly smaller than in Fig 7B, yet the IOU metric does not differentiate the proximity of the frames. In scenarios (c) and (d), where frame A intersects with frame B, the unchanged IOU value fails to indicate the degree of intersection between the two bounding boxes.

In contrast, DIOU incorporates the distance, overlap, and scale between two frames. It effectively reflects the relative positioning between the predicted and target frames and enables direct comparison of the distances when the images are disjoint. The formulas for DIOU calculation are presented below:

$$S_{DIOU}=S_{IOU}-\frac{\rho^{2}(b,b^{gt})}{c^{2}}$$ (2)

$$L_{DIOU}=1-S_{IOU}+\frac{\rho^{2}(b,b^{gt})}{c^{2}}$$ (3)

Where b and *b^gt^* represent the central points of the detection and prediction bounding boxes, respectively, *ρ* denotes the Euclidean distance between these central points, c is the diagonal of the minimal enclosing rectangle, and *L_DIOU_* is the DIOU loss function. In this study, DIOU replaces IOU to more accurately measure the match between detected and predicted frames, thereby enhancing the tracking system’s efficiency and robustness.

The improved algorithm’s procedure is described as follows:

1. The trajectory from the previous iteration is predicted by the KF to determine the mean and covariance for the current round. The statuses of confirmed and unconfirmed data remain unchanged.
2. The trajectory from step (1) and the detection from the current target detection round undergo cascade matching. The outcomes are categorized into mismatched trajectory, mismatched detection, and matched trajectory.
3. Detection omissions in step (2) are combined with undetermined trajectories from step (1) and matched again using DIOU, resulting in more reliable mismatch traces, mismatch detections, and matching trajectories.
4. For unmatched trajectories where the unconfirmed and confirmed statuses have aged beyond the predefined threshold, the status is set to ’delete’.
5. This step involves the output of results and preparation for the next round. Matching trajectories from steps (3) and (4) are merged to update the KF, and ages are incremented by one for the output. Additionally, a trajectory is created for the unmatched detection result from step (3), and trajectories are created for those below the age threshold confirmed in step (4). Finally, trajectories from these three sources are consolidated into the output for the current round and prepared as input for the next round, returning to step (1).

## 4. Experiments results and discussion

### 4.1 Experiment setups

The experimental setup utilized the NVIDIA GeForce RTX 4070 graphics card, supported by CUDA 11.1 and CUDNN-V8.0.4.30 GPU acceleration libraries. The computational environment was configured with PyTorch version 1.8.1 and Python 3.8 to support the development of a detection model characterized by high stability and rapid convergence. The hardware specifications are detailed in Table 2.

**Table 2:** Experimental environment.

Name	Configuration information
Operating system	Windows 10
Graphics card	NVIDIA GeForce RTK 4070
CPU	AMD Ryzen 9 5900X
Software	Python 3.8, Pycharm 2020.1

### 4.2 Evaluation indicators

The performance of the algorithm was assessed using several evaluation metrics. The model was tasked with detecting multiple target classes, each capable of generating a PR curve. From these curves, the Average Precision (AP) for each class was calculated, which represents the area under the PR curve. The mAP was computed as the average of the AP values across all classes, as shown in Eq (4):

$$mAP=\frac{1}{class\_number}\sum_{1}^{class\_number}\;AP$$ (4)

MOT accuracy (MOTA) was used as a measure of tracking performance, emphasizing the algorithm’s ability to detect objects and maintain accurate trajectories, independent of the positional accuracy of the detected objects. Higher MOTA values indicate better tracking performance, calculated according to Eq (5):

$$MOTA=1-\frac{\sum tFN+FP+IDSW}{\sum tGT_{t}}$$ (5)

MOT precision (MOTP) was used to gauge the positional accuracy of the tracking, where a higher MOTP value indicates greater accuracy. This metric is calculated as shown in Eq (6):

$$MOTP=\frac{\sum t,id_{t,i}}{\sum tc_{t}}$$ (6)

FP represents the count of erroneous alerts pertaining to the trajectory of incorrectly predicted objects.

FN represents the total number of objects that are missed by the detection and tracking systems.

IDs indicates that the frequency of assigning IDs has changed.

### 4.3 Evaluation of benchmarks

#### 4.3.1 Transfer learning results

**Fig 8:**
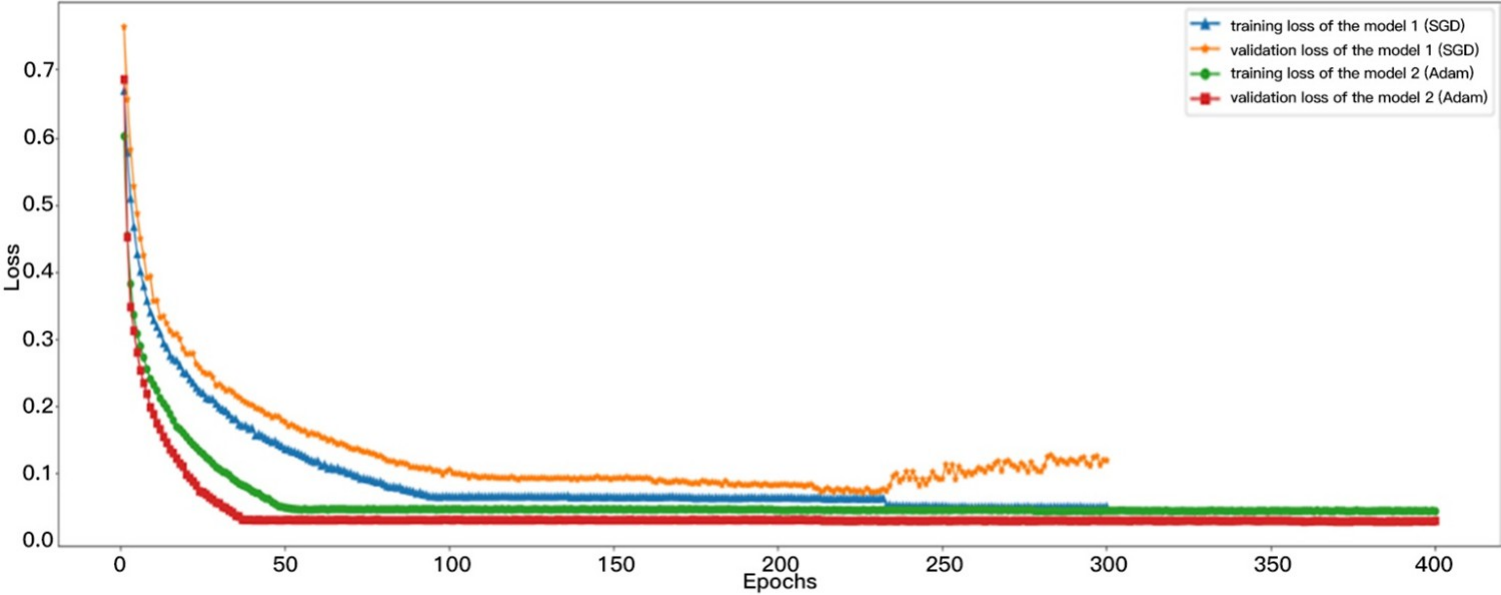
Curves of training and validation losses during model pre-training.

The loss function utilized for pre-training, employing datasets and models 1 and 2, is depicted in Fig 8. The results demonstrate that the validation loss associated with the SGD optimizer began to rise after the 230th epoch, signaling a gradual onset of overfitting in model 1. Initially, both the training and validation losses decreased significantly and then plateaued, completing their trajectories under the Adam algorithm.

**Fig 9:**
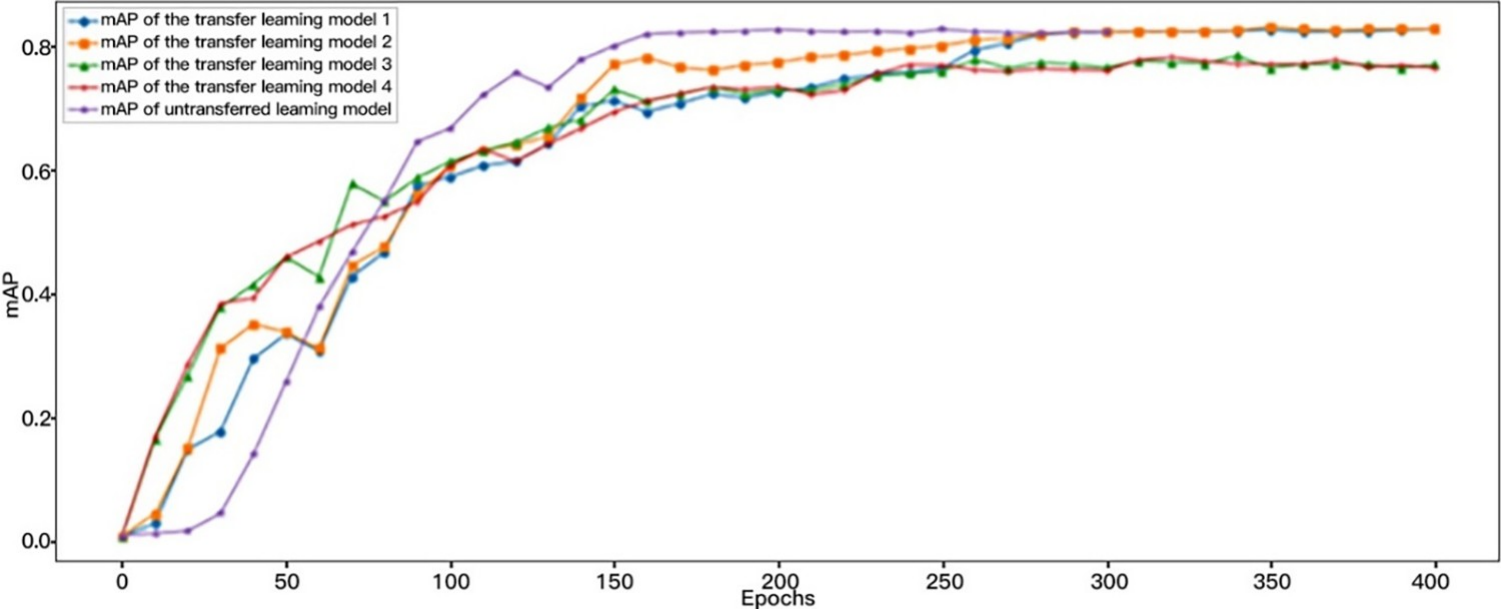
mAP values of the transfer learning models and the un-transferred learning model.

In scenarios with limited datasets, transfer learning can be leveraged to train models, optimizing the model structure and enhancing performance, as well as improving the mean Average Precision (mAP) of the models. Fig 9 illustrates a comparison of the mAP scores for models with and without iterative weight application through transfer learning. Owing to the gradient descent method utilized by the Adam optimizer, the weights in transfer learning models 3 and 4 show a bias towards the publicly available ship dataset. This bias results in suboptimal feature extraction from these models, adversely affecting their mAP values. Conversely, the mAP values for transfer learning models 1 and 2 reached a notable high of 0.82, affirming the efficacy of these models. This outcome also suggests that pre-training with appropriate transfer learning models can significantly enhance the detection accuracy.

**Fig 10:**
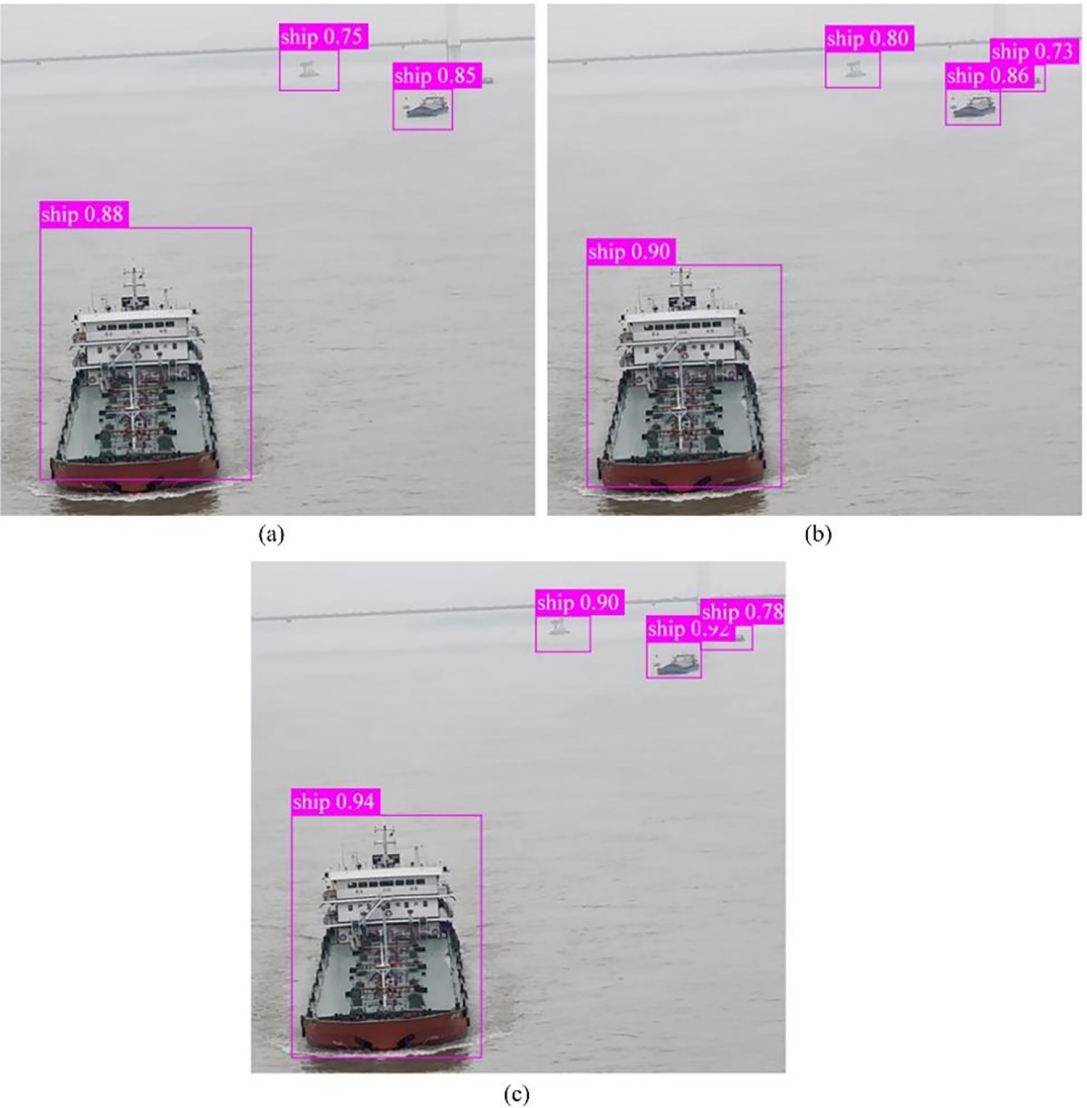
Results of different models for ship detection. (a) Non-transfer learning model. (b) Transfer learning model 1. (c) Transfer learning model 2.

Fig 10 presents a comparison of ship detection results between pre-trained models using transfer learning models 1 and 2, and those without transfer learning. As illustrated, pre-training with transfer learning models demonstrates higher detection accuracy and confidence near detection frames, and can reduce the occurrence of missed detections. Furthermore, compared to using transfer learning model 1, transfer learning model 2 exhibited higher detection confidence, validating its suitability for model pre-training in this study.

Although transfer learning model 2 has addressed some issues of missed detections, it still encounters challenges in distinguishing two adjacent vessels as separate entities. To address this issue, enhancements to the YOLOv7 model have been introduced. Experimental verification of these improvements is discussed in the subsequent sections.

#### 4.3.2 Detection results

This study introduces a custom dataset specifically tailored for ship detection tasks, which has been systematically divided into training, validation, and testing sets to facilitate thorough model evaluation. The YOLOv7 model used in this research incorporates the SimAM attention mechanism and employs the PConv to optimize the multi-scale feature fusion module. These enhancements not only improve detection speed but also reduce the model’s parameters and computational burden, leading to the development of an advanced YOLOv7 variant. A comprehensive performance evaluation was conducted to compare the Enhanced YOLOv7 model with other prominent models such as SSD, YOLOv5, YOLOv7, and YOLOv8. The results, as depicted in Table 3, clearly demonstrate the superior performance of the Enhanced YOLOv7 model, which, along with the other YOLOv7 variants, was trained using transfer learning techniques.

**Table 3:** Comparison with different detection methods.

Model	mAP↑(%)	Parameters↑(M)	FPS↑
SSD	84.2	210	165
YOLOv5	89.6	13.9	135
YOLOv7	91.0	12.8	156
YOLOv8	91.8	30.6	112
Ours	94.1	11.3	168

Table 3 indicates that our model outperforms competitors such as YOLOv8. This improvement is attributable to the integration of the SimAM and PConv modules. The integration of the SimAM attention mechanism into the YOLOv7 detection framework significantly enhances the model’s focus on relevant image features, thereby improving the accuracy of object detection. Furthermore, the introduction of PConv optimizes the multi-scale feature fusion module, enhancing detection speed while simultaneously reducing both the number of parameters and the computational load. Compared with the original YOLOv7 model, our model not only achieves a higher detection accuracy, with an improvement of 3.1%, but also demonstrates significant advancements in terms of a reduced parameter count (11.3M) and an increased frame rate (168 FPS). These enhancements further validate the effectiveness of the enhanced features integrated into our model.

#### 4.3.3 Tracking results

To assess the innovation and robustness of our proposed UAV-based MOT system for monitoring multiple vessels, we conducted a comparative analysis with several mainstream multi-target tracking methods. These benchmark methods employed the original YOLOv7 model for detection, whereas our approach utilized an enhanced YOLOv7 model. This modification was intended to underscore the superior effectiveness of our integrated system design. As illustrated in Table 4, our evaluation encompassed a variety of popular tracking algorithms, including MOTDT, SORT, Deep SORT, ByteTrack, and BoT-SORT, showcasing the comparative advantages of our method.

**Table 4:** Comparison of tracking results with other mainstream methods.

Tracker	Detector	MOTA↑(%)	MOTP↑(%)	FP↓	FN↓	IDs↓
MOTDT	YOLOv7	43.2	68.3	6052	4281	579
SORT	YOLOv7	49.6	72.3	4863	4037	741
Deep SORT	YOLOv7	58.4	78.9	3256	2732	385
ByteTrack	YOLOv7	60.1	79.6	4326	2968	402
BoT-SORT	YOLOv7	62.5	81.2	3518	2376	351
Ours	Improved YOLOv7	65.3	81.9	2689	2062	253

Ship monitoring poses significant challenges due to occlusions and the complexity of the scenes, which necessitates an advanced model capable of operating under constrained computational resources and challenging tracking conditions. As detailed in Table 4, our model achieves an MOTA of 65.3%, an MOTP of 81.9%, with 2689 FP, 2062 FN, and 253 IDs. Compared to the baseline model, Deep SORT, our model demonstrates improvements with a 6.9% increase in MOTA, a 3.0% increase in MOTP, 567 fewer FPs, 670 fewer FNs, and 132 fewer IDs, thereby proving the superiority of our model. Moreover, when compared with other mainstream models such as BoT-SORT, our model exhibits the best overall performance, further validating its reliability for tracking in complex environments.

**Fig 11:**
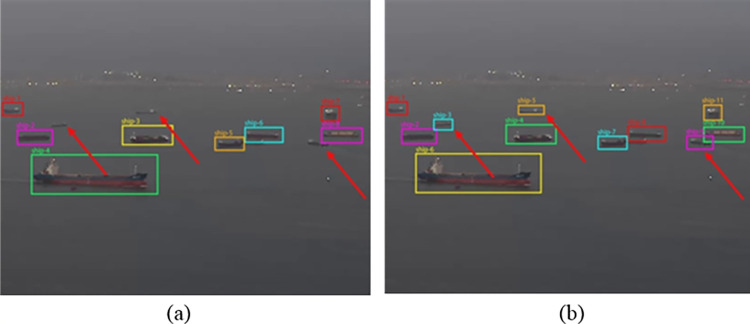
Results of different algorithms for ship detection and tracking. (a) YOLOv7 + Deep SORT. (b) Ours.

This study utilized an enhanced YOLOv7 algorithm for object detection paired with an improved DeepSORT algorithm for tracking. The comparative tracking results, depicted in Fig 11, illustrate the target tracking scenarios. Fig 11A and 11B highlight situations where, due to the small size of some targets, the original YOLOv7 and DeepSORT algorithms fail to detect and track them, as indicated by the red arrows. In contrast, the optimized YOLOv7 and DeepSORT algorithms successfully detect and track these targets. This success is primarily due to three factors: (1) model pre-training using transfer learning techniques, which enhances the detection model’s ability to recognize more ship features and improves its detection capabilities; (2) the integration of the SimAM attention mechanism and PConv convolution, which further boosts the detection capability of the model; (3) the introduction of the Distance-IoU (DIOU) function during the IOU matching stage of DeepSORT, effectively reducing missed detections caused by target aggregation or occlusion. These visual tracking results further underscore the effectiveness and robustness of our enhancements.

### 4.4 Ablation experiment

**Fig 12:**
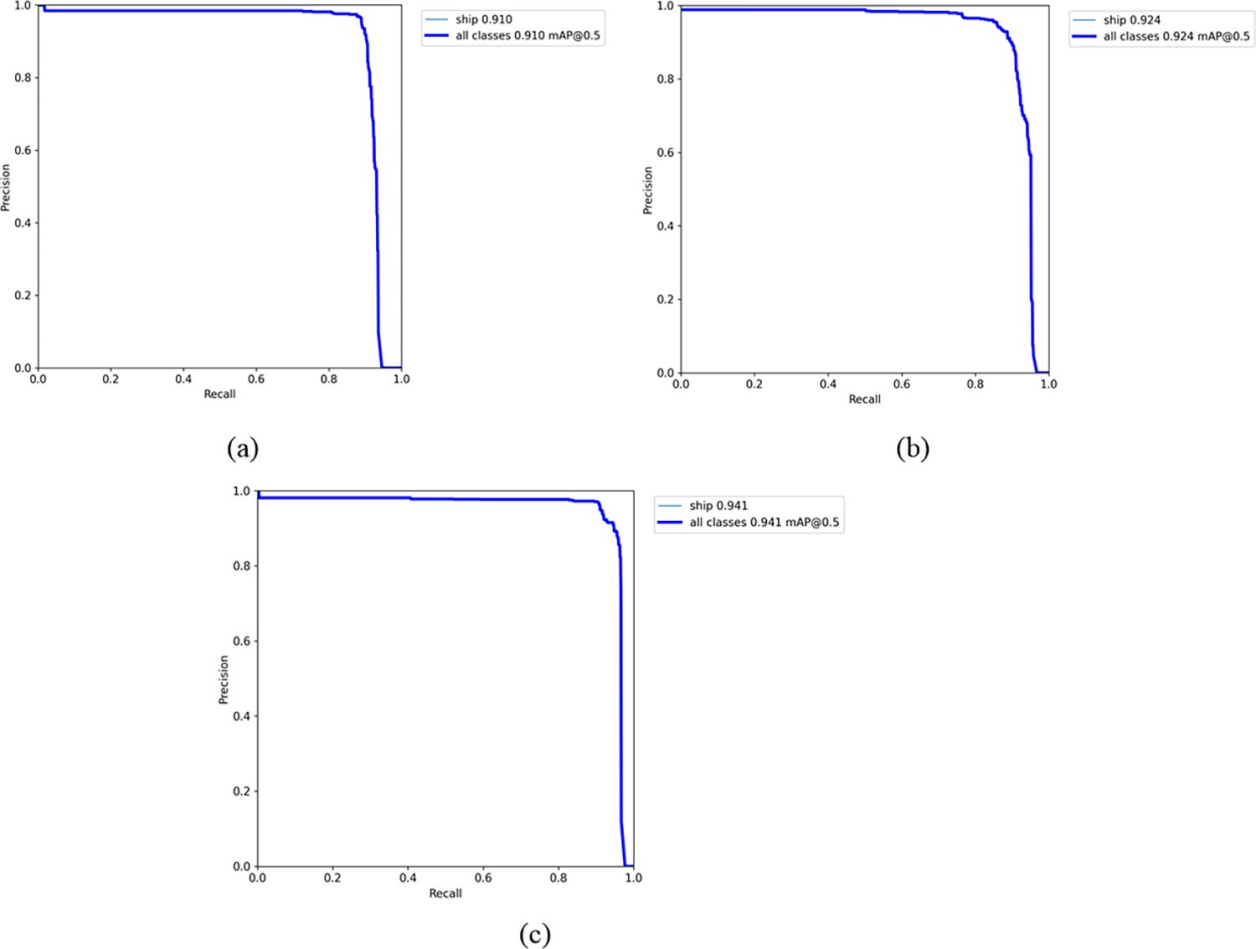
Improvement of ablation experiment results for YOLOv7 modules. (a) The original YOLO model (b)YOLOv7+SimAM. (c)YOLOv7+SimAM+Pconv.

To verify the effectiveness of each enhancement introduced in our modules, we conducted a series of ablation experiments. Initially, we evaluated the impact of improvements made to the YOLOv7 modules. As shown in Fig 12, the original YOLOv7 model demonstrated an mAP of 91%. After integrating the SimAM attention mechanism, the mAP increased to 92.4%. With the subsequent addition of the PConv module, the model achieved its highest mAP of 94.1%. These results confirm the individual and combined effectiveness of each enhancement in the detection model, indicating that both the SimAM attention mechanism and the PConv module significantly boost the mAP without any compatibility issues.

To further validate the performance enhancements attributable to the modules introduced in this study, we conducted detailed ablation studies using the YOLOv7 and Deep SORT algorithms, as represented in Table 5. These experiments were meticulously designed to quantify the contributions of each new module towards enhancing the accuracy and robustness of ship tracking systems under various environmental conditions.

**Table 5:** Ablation study of different improved methods.

Baseline	SimAM	Pconv	DIOU	MOTA↑(%)	MOTP↑(%)	IDs↓
√				58.4	78.9	385
√	√			60.1	79.8	356
√	√	√		62.8	81.3	305
√	√	√	√	65.3	81.9	253

As depicted in Table 5, the integration of the SimAM attention mechanism and PConv module notably enhances the accuracy of the MOT. By incorporating the SimAM attention mechanism, attention weights are applied to the spatial positions of feature maps, enabling the model to more effectively capture significant local features. After introducing the SimAM attention mechanism, the MOTA and MOTP of the model improved by 1.7% and 0.9%, respectively. Furthermore, the PConv module aids the model in focusing on effective areas within the image when dealing with occlusion, thus mitigating interference and enhancing the detection capabilities for targets. The integration of PConv led to an increase in MOTA by 2.7% and MOTP by 1.5%. These enhancements underscore the critical role of advanced detectors within the TBD-based tracking system framework. Additionally, incorporating the DIOU function significantly refines various performance metrics related to tracking accuracy and reduces the occurrence of missed detections. Notably, the introduction of DIOU resulted in a 2.5% improvement in MOTA, a 0.6% increase in MOTP, and a reduction of 52 identity switches. These findings demonstrate that replacing the IOU function with DIOU in the matching stage of Deep SORT effectively amplifies tracking performance, particularly in challenging scenarios involving small targets and occlusion.

## 5. Conclusion

This study introduced a reliable UAV MOT system for ships monitoring, which utilized improved YOLOv7 and Deep SORT to enhance the accuracy of multi-ship tracking. Specifically, it used transfer learning to train YOLOv7 models, effectively solving the problem of insufficient training data. Meanwhile, by incorporating the SimAM attention mechanism and PConv convolution into the YOLOv7 model, the accuracy of object detection was improved, and the number of parameters and computational workload were reduced. In addition, in the IOU matching stage of the Deep SORT algorithm, replacing the IOU with DIOU improved the target trajectory and matching accuracy, which enhanced the tracking performance and robustness of the system. Compared with the original YOLOv7 and Deep SORT algorithms, this system improved MOTA and MOTP by 6.9% and 3.0%, respectively, verifying its effectiveness. Comprehensive analysis showed that the proposed system was effective for ship monitoring tasks, and intelligent drones using this method could prevent ship collisions in waterways through real-time monitoring. This was of great significance and potential for strengthening ship monitoring efforts.

In future research, we will aim to further reduce the number of parameters and computational complexity of the model to enhance its suitability for edge inference platforms. Additionally, we will explore the integration of global motion compensation into the MOT algorithm, which is an effective image alignment technique for addressing background motion. This method involves translational suppression of local outliers, extraction of image key points, and sparse optical flow for feature tracking. By compensating for the motion of the UAV camera, this approach will enhance the overall effectiveness of the MOT system.

## References

[1] Xu F., Liu J., Dong C., & Wang X. (2017). Ship detection in optical remote sensing images based on wavelet transform and multi-level false alarm identification. Remote Sensing, 9(10), 985.

[2] Bo L. I., Xiaoyang X. I. E., Xingxing W. E. I., & Wenting T. A. N. G. (2021). Ship detection and classification from optical remote sensing images: A survey. Chinese Journal of Aeronautics, 34(3), 145–163.

[3] Yu W., You H., Lv P., Hu Y., & Han B. (2021). A moving ship detection and tracking method based on optical remote sensing images from the geostationary satellite. Sensors, 21(22), 7547. doi: 10.3390/s21227547 34833622 PMC8619672

[4] Xu Q., Li Y., Zhang M., & Li W. (2022). COCO-Net: A dual-supervised network with unified ROI-loss for low-resolution ship detection from optical satellite image sequences. IEEE Transactions on Geoscience and Remote Sensing, 60, 1–15.

[5] Teixeira E., Araujo B., Costa V., Mafra S., & Figueiredo F. (2022). Literature review on ship localization, classification, and detection methods based on optical sensors and neural networks. Sensors, 22(18), 6879. doi: 10.3390/s22186879 36146228 PMC9501387

[6] Han Y., Guo J., Yang H., Guan R., & Zhang T. (2024). SSMA-YOLO: A Lightweight YOLO Model with Enhanced Feature Extraction and Fusion Capabilities for Drone-Aerial Ship Image Detection. Drones, 8(4), 145.

[7] Lu Z., Wang P., Li Y., & Ding B. (2023). A New Deep Neural Network Based on SwinT-FRM-ShipNet for SAR Ship Detection in Complex Near-Shore and Offshore Environments. Remote Sensing, 15(24), 5780.

[8] Tian Y., Wang X., Zhu S., Xu F., & Liu J. (2023). LMSD-Net: a lightweight and high-performance ship detection network for optical remote sensing images. Remote Sensing, 15(17), 4358.

[9] Wu J., Cao C., Zhou Y., Zeng X., Feng Z., Wu Q., & Huang Z. (2021). Multiple ship tracking in remote sensing images using deep learning. Remote Sensing, 13(18), 3601.

[10] Song R., Li T., & Li T. (2023). Ship detection in haze and low-light remote sensing images via colour balance and DCNN. Applied Ocean Research, 139, 103702.

[11] Li J., Li Z., Chen M., Wang Y., & Luo Q. (2022). A new ship detection algorithm in optical remote sensing images based on improved R3Det. Remote Sensing, 14(19), 5048.

[12] Han J., Ding J., Li J., & Xia G. S. (2021). Align deep features for oriented object detection. IEEE transactions on geoscience and remote sensing, 60, 1–11.

[13] Zhang M., Rong X., & Yu X. (2022). Light-SDNet: a lightweight CNN architecture for ship detection. IEEE Access, 10, 86647–86662.

[14] Zhang, L., Wang, Y., & Chen, W. (2021, December). ARS-Det: an axis-based anchor-free rotation detector for ship in remote sensing images. In International Conference on Environmental Remote Sensing and Big Data (ERSBD 2021) (Vol. 12129, pp. 15–23). SPIE.

[15] Zhao J., Chen Y., Zhou Z., Zhao J., Wang S., & Chen X. (2022). Extracting vessel speed based on machine learning and drone images during ship traffic flow prediction. Journal of Advanced Transportation, 2022(1), 3048611.

[16] Zhao J., Chen Y., Zhou Z., Zhao J., Wang S., & Chen X. (2023). Multiship speed measurement method based on machine vision and drone images. IEEE Transactions on Instrumentation and Measurement, 72, 1–12.37323850

[17] Chen X., Xu X., Yang Y., Wu H., Tang J., & Zhao J. (2020). Augmented ship tracking under occlusion conditions from maritime surveillance videos. IEEE Access, 8, 42884–42897.

[18] Hu, Y., Yang, C., Yang, J., Li, Y., Jing, W., & Shu, S. (2021, July). Review on unmanned aerial vehicle remote sensing and its application in coastal ecological environment monitoring. In IOP Conference Series: Earth and Environmental Science (Vol. 821, No. 1, p. 012018). IOP Publishing.

[19] Wu X., Li W., Hong D., Tao R., & Du Q. (2021). Deep learning for unmanned aerial vehicle-based object detection and tracking: A survey. IEEE Geoscience and Remote Sensing Magazine, 10(1), 91–124.

[20] Chen X., Tang J., & Lao S. (2020). Review of unmanned aerial vehicle swarm communication architectures and routing protocols. Applied Sciences, 10(10), 3661.

[21] Wang Q., Wang J., Wang X., Wu L., Feng K., & Wang G. (2024). A YOLOv7-based method for ship detection in videos of drones. Journal of Marine Science and Engineering, 12(7), 1180.

[22] Han Y., Guo J., Yang H., Guan R., & Zhang T. (2024). SSMA-YOLO: A Lightweight YOLO Model with Enhanced Feature Extraction and Fusion Capabilities for Drone-Aerial Ship Image Detection. Drones, 8(4), 145.

[23] Li Z., Deng Z., Hao K., Zhao X., & Jin Z. (2024). A Ship Detection Model Based on Dynamic Convolution and an Adaptive Fusion Network for Complex Maritime Conditions. Sensors, 24(3), 859. doi: 10.3390/s24030859 38339576 PMC10856874

[24] Li Y., Yuan H., Wang Y., & Xiao C. (2022). GGT-YOLO: A novel object detection algorithm for drone-based maritime cruising. Drones, 6(11), 335.

[25] Chen Z., Chen D., Zhang Y., Cheng X., Zhang M., & Wu C. (2020). Deep learning for autonomous ship-oriented small ship detection. Safety Science, 130, 104812.

[26] Zhao H., Zhang H., & Zhao Y. (2023). Yolov7-sea: Object detection of maritime uav images based on improved yolov7. In Proceedings of the IEEE/CVF winter conference on applications of computer vision (pp. 233–238).

[27] Shan Y., Zhou X., Liu S., Zhang Y., & Huang K. (2020). SiamFPN: A deep learning method for accurate and real-time maritime ship tracking. IEEE Transactions on Circuits and Systems for Video Technology, 31(1), 315–325.

[28] Yang X., Wang Y., Wang N., & Gao X. (2021). An enhanced SiamMask network for coastal ship tracking. IEEE Transactions on Geoscience and Remote Sensing, 60, 1–11.

[29] Wojke, N., Bewley, A., & Paulus, D. (2017, September). Simple online and realtime tracking with a deep association metric. In 2017 IEEE international conference on image processing (ICIP) (pp. 3645–3649). IEEE.

[30] Bewley, A., Ge, Z., Ott, L., Ramos, F., & Upcroft, B. (2016, September). Simple online and realtime tracking. In 2016 IEEE international conference on image processing (ICIP) (pp. 3464–3468). IEEE.

[31] Aharon, N., Orfaig, R., & Bobrovsky, B. Z. (2022). BoT-SORT: Robust associations multi-pedestrian tracking. arXiv preprint arXiv:2206.14651.

[32] Aharon, N., Orfaig, R., & Bobrovsky, B. Z. (2022). BoT-SORT: Robust associations multi-pedestrian tracking. arXiv preprint arXiv:2206.14651.

[33] Zhang W., He X., Li W., Zhang Z., Luo Y., Su L., & Wang P. (2021). A robust deep affinity network for multiple ship tracking. IEEE Transactions on Instrumentation and Measurement, 70, 1–20.33776080

[34] Tang G., Liu S., Fu**o I., Claramunt C., Wang Y., & Men S. (2020). H-YOLO: A single-shot ship detection approach based on region of interest preselected network. Remote Sensing, 12(24), 4192.

[35] Jiang J., Fu X., Qin R., Wang X., & Ma Z. (2021). High-speed lightweight ship detection algorithm based on YOLO-v4 for three-channels RGB SAR image. Remote Sensing, 13(10), 1909.

[36] Xu X., Zhang X., & Zhang T. (2022). Lite-yolov5: A lightweight deep learning detector for on-board ship detection in large-scene sentinel-1 sar images. Remote Sensing, 14(4), 1018.

[37] Yao, Z., Chen, X., & Shi, C. (2023, May). Research on Surface Environment Perception via Camera-LiDAR Sensor Fusion. In 2023 6th International Conference on Artificial Intelligence and Big Data (ICAIBD) (pp. 895–899). IEEE.

[38] Zhao, H., Zhang, H., & Zhao, Y. (2023). Yolov7-sea: Object detection of maritime uav images based on improved yolov7. In Proceedings of the IEEE/CVF winter conference on applications of computer vision (pp. 233–238).

[39] Li, C., Zhou, A., & Yao, A. (2022). Omni-dimensional dynamic convolution. arxiv preprint arxiv:2209.07947.

[40] Cheng S., Zhu Y., & Wu S. (2023). Deep learning based efficient ship detection from drone-captured images for maritime surveillance. Ocean Engineering, 285, 115440.

[41] Zhao X., & Song Y. (2023). Improved ship detection with YOLOv8 enhanced with MobileViT and GSConv. Electronics, 12(22), 4666.

[42] Park H., Ham S. H., Kim T., & An D. (2022). Object recognition and tracking in moving videos for maritime autonomous surface ships. Journal of Marine Science and Engineering, 10(7), 841.

[43] Han J., Cho Y., Kim J., Kim J., Son N. S., & Kim S. Y. (2020). Autonomous collision detection and avoidance for ARAGON USV: Development and field tests. Journal of Field Robotics, 37(6), 987–1002.

[44] Lee W. J., Roh M. I., Lee H. W., Ha J., Cho Y. M., Lee S. J., & Son N. S. (2021). Detection and tracking for the awareness of surroundings of a ship based on deep learning. Journal of Computational Design and Engineering, 8(5), 1407–1430.

[45] Liu Y., Liu Y., Zhong Z., Chen Y., **a, J., & Chen, Y. (2023). Depth tracking of occluded ships based on SIFT feature matching. KSII Trans. Internet Inf. Syst., 17(4), 1066–1079.

[46] Ding H., & Weng J. (2024). A robust assessment of inland waterway collision risk based on AIS and visual data fusion. Ocean Engineering, 307, 118242.

[47] Guo Y., Shen Q., Ai D., Wang H., Zhang S., & Wang X. (2024). Sea-IoUTracker: A more stable and reliable maritime target tracking scheme for unmanned vessel platforms. Ocean Engineering, 299, 117243.

[48] Guo Y., Liu R. W., Qu J., Lu Y., Zhu F., & Lv Y. (2023). Asynchronous trajectory matching-based multimodal maritime data fusion for vessel traffic surveillance in inland waterways. IEEE Transactions on Intelligent Transportation Systems, 24(11), 12779–12792.

[49] Wang Q., Wang J., Wang X., Wu L., Feng K., & Wang G. (2024). A YOLOv7-based method for ship detection in videos of drones. Journal of Marine Science and Engineering, 12(7), 1180.

[50] Wang Q., Wang J., Wang X., Wu L., Feng K., & Wang G. (2024). A YOLOv7-based method for ship detection in videos of drones. Journal of Marine Science and Engineering, 12(7), 1180.

[51] Wang, C. Y., Bochkovskiy, A., & Liao, H. Y. M. (2023). YOLOv7: Trainable bag-of-freebies sets new state-of-the-art for real-time object detectors. In Proceedings of the IEEE/CVF conference on computer vision and pattern recognition (pp. 7464–7475).

[52] Gao, P., Lu, J., Li, H., Mottaghi, R., & Kembhavi, A. (2021). Container: Context aggregation network. arXiv preprint arXiv:2106.01401.

[53] Dollár, P., Singh, M., & Girshick, R. (2021). Fast and accurate model scaling. In Proceedings of the IEEE/CVF Conference on Computer Vision and Pattern Recognition (pp. 924–932).

[54] Vasu, P. K. A., Gabriel, J., Zhu, J., Tuzel, O., & Ranjan, A. (2023). Mobileone: An improved one millisecond mobile backbone. In Proceedings of the IEEE/CVF conference on computer vision and pattern recognition (pp. 7907–7917).

[55] Zhang, X., Zeng, H., Guo, S., & Zhang, L. (2022, October). Efficient long-range attention network for image super-resolution. In European conference on computer vision (pp. 649–667). Cham: Springer Nature Switzerland.

[56] Lee, Y., Hwang, J. W., Lee, S., Bae, Y., & Park, J. (2019). An energy and GPU-computation efficient backbone network for real-time object detection. In Proceedings of the IEEE/CVF conference on computer vision and pattern recognition workshops (pp. 0–0).

[57] Wang, C. Y., Liao, H. Y. M., Wu, Y. H., Chen, P. Y., Hsieh, J. W., & Yeh, I. H. (2020). CSPNet: A new backbone that can enhance learning capability of CNN. In Proceedings of the IEEE/CVF conference on computer vision and pattern recognition workshops (pp. 390–391).

[58] Chen, J., Kao, S. H., He, H., Zhuo, W., Wen, S., Lee, C. H., & Chan, S. H. G. (2023). Run, don’t walk: chasing higher FLOPS for faster neural networks. In Proceedings of the IEEE/CVF conference on computer vision and pattern recognition (pp. 12021–12031).

